# Genomics Reveals the Worldwide Distribution of Multidrug-Resistant Serotype 6E Pneumococci

**DOI:** 10.1128/JCM.00744-15

**Published:** 2015-06-18

**Authors:** Andries J. van Tonder, James E. Bray, Lucy Roalfe, Rebecca White, Marta Zancolli, Sigríður J. Quirk, Gunnsteinn Haraldsson, Keith A. Jolley, Martin C. J. Maiden, Stephen D. Bentley, Ásgeir Haraldsson, Helga Erlendsdóttir, Karl G. Kristinsson, David Goldblatt, Angela B. Brueggemann

**Affiliations:** a Nuffield Department of Medicine, University of Oxford, Oxford, United Kingdom; b Department of Zoology, University of Oxford, Oxford, United Kingdom; c Institute of Child Health, University College London, London, United Kingdom; d University of Iceland, Reykjavik, Iceland; e Landspitali University Hospital, Reykjavik, Iceland; f Pathogen Genomics, Wellcome Trust Sanger Institute, Hinxton, United Kingdom

## Abstract

The pneumococcus is a leading pathogen infecting children and adults. Safe, effective vaccines exist, and they work by inducing antibodies to the polysaccharide capsule (unique for each serotype) that surrounds the cell; however, current vaccines are limited by the fact that only a few of the nearly 100 antigenically distinct serotypes are included in the formulations. Within the serotypes, serogroup 6 pneumococci are a frequent cause of serious disease and common colonizers of the nasopharynx in children. Serotype 6E was first reported in 2004 but was thought to be rare; however, we and others have detected serotype 6E among recent pneumococcal collections. Therefore, we analyzed a diverse data set of ∼1,000 serogroup 6 genomes, assessed the prevalence and distribution of serotype 6E, analyzed the genetic diversity among serogroup 6 pneumococci, and investigated whether pneumococcal conjugate vaccine-induced serotype 6A and 6B antibodies mediate the killing of serotype 6E pneumococci. We found that 43% of all genomes were of serotype 6E, and they were recovered worldwide from healthy children and patients of all ages with pneumococcal disease. Four genetic lineages, three of which were multidrug resistant, described ∼90% of the serotype 6E pneumococci. Serological assays demonstrated that vaccine-induced serotype 6B antibodies were able to elicit killing of serotype 6E pneumococci. We also revealed three major genetic clusters of serotype 6A capsular sequences, discovered a new hybrid 6C/6E serotype, and identified 44 examples of serotype switching. Therefore, while vaccines appear to offer protection against serotype 6E, genetic variants may reduce vaccine efficacy in the longer term because of the emergence of serotypes that can evade vaccine-induced immunity.

## INTRODUCTION

The pneumococcus (Streptococcus pneumoniae) is one of the most important pathogens worldwide. An estimated 1.3 million children die every year from pneumonia, and the pneumococcus is the leading cause (1, 2). It is also a leading cause of death due to bacteremia and meningitis among young children and is a major cause of disease among adults, particularly the elderly, among whom there is also a high risk of death (3, 4). Pneumococcal conjugate vaccines (PCVs) are administered to children in many developed and resource-poor countries and have been an enormous public health success, significantly reducing morbidity and mortality in the countries that have implemented widespread vaccination (5, 6).

Pneumococci are differentiated by an antigenic polysaccharide capsule (“serotype”) that surrounds the cell and protects the pneumococcus from being phagocytosed by the human immune system. The polysaccharide capsule is an essential pneumococcal virulence factor and forms the basis for PCV-mediated protection against pneumococcal disease. The first PCV (PCV7) was licensed in 2000 and included seven serotypes: 4, 6B, 9V, 14, 18C, 19F, and 23F (7). PCV7 was later superseded by PCV13, which added serotypes 1, 3, 5, 6A, 7F, and 19A to the original PCV7 (8), and PCV10, which contains the original PCV7 serotypes plus serotypes 1, 5, and 7F (9).

However, nearly 100 different serotypes have been characterized and new ones continue to be discovered (10–13). Current PCV formulations have limited serotype coverage, and their use has been associated with a significantly altered serotype distribution. Disease due to vaccine serotype pneumococci decreases, but an increase in the proportion of disease caused by nonvaccine serotype pneumococci has been observed, although there is heterogeneity in this serotype replacement disease phenomenon that is not well understood (14, 15). Furthermore, the prevalence of commensal (carriage) pneumococci in the nasopharynx, its ecological niche, generally remains the same after PCV but reorders in favor of nonvaccine types (16). Vaccine escape is also possible, and new genetic variants can spread rapidly (17–19). Consequently, protection from pneumococcal disease remains a challenge.

Apart from two known exceptions (serotypes 3 and 37), the polysaccharide capsule is synthesized by the Wzx/Wzy-dependent pathway and the associated genes are located in the capsular polysaccharide synthesis (*cps*) locus. The majority of the genes in the *cps* locus are present in all *cps* loci, and there are three genes in particular, *wciP*, *wzy*, and *wzx*, that have serotype-specific alleles (20, 21). Horizontal genetic exchange of all or part of the *cps* locus sequence, between related and unrelated pneumococcal lineages, has been well documented (17, 18, 22–25).

Serogroup 6 is a particularly important serogroup, as it is one of the most common serotypes found in the nasopharynxes of unvaccinated young children and is a major cause of serious pneumococcal disease among all age groups (5, 26). Serotypes 6A and 6B have been recognized for many decades, but more recently, serotypes 6C and 6D, which are genetically similar to serotypes 6A and 6B, were discovered (27–29). From a vaccine perspective, it was shown that the serotype 6B antibodies elicited by PCV7 were partially protective against serotype 6A but not serotype 6C (15, 30, 31). However, PCV13-induced antibodies were shown to elicit killing of serotypes 6A, 6B, and 6C (31, 32) and PCV10-induced antibodies mediated the killing of serotype 6B and possibly serotype 6A (15, 33). Serotype 6D pneumococci have been reported infrequently, although their prevalence in South Korea was estimated to be 10%, and a PCV7-induced cross-protective immune response to serotype 6D was demonstrated (34, 35). Serotypes 6F, 6G, and 6H have been described very recently, and whether PCVs provide any protection against these serotypes is unknown (10, 13).

The first report of serotype 6E pneumococci was by Mavroidi et al., whose study explored sequence diversity and evolution among serogroup 6 pneumococci. Internal fragments of the three serotype-specific genes were sequenced in a diverse collection of 102 isolates of serotype 6A and 6B pneumococci. While they found little sequence divergence between serotype 6A and most of the serotype 6B isolates, they did identify a group of what they called “class 2” serotype 6B sequences, which were >5% divergent from the majority of serotype 6B isolates (36). Two subsequent studies of serogroup 6 diversity and evolution in other pneumococcal collections confirmed the existence of “class 2” serotype 6B or what one report called “6B-III” or possible “serotype 6E” strains (29, 37). Very recently, investigators have reported serotype 6E pneumococci in several Asian countries (38–40). As part of an ongoing vaccine impact study characterizing Icelandic pneumococci pre- and postimplementation of PCV10, we also discovered serotype 6E strains. Furthermore, we interrogated the genome sequences of several serotype 6B Pneumococcal Molecular Epidemiology Network (PMEN) reference strains and found that they all possessed a serotype 6E *cps* locus sequence. As far as we are aware, the biochemical structure of serotype 6E polysaccharide is not known.

Therefore, we compiled and investigated a large and diverse data set of ∼1,000 serogroup 6 pneumococcal genomes with three aims: (i) to determine the prevalence, distribution, and epidemiology of serotype 6E (as defined by the *cps* locus sequence); (ii) to examine the genetics of the serogroup 6 *cps* locus and the molecular epidemiology of serogroup 6 lineages; and (iii) to assess whether the serotype 6B polysaccharides in PCV7 and PCV13 induce the production of protective antibodies to serotype 6E.

## MATERIALS AND METHODS

### Pneumococcal genome data set.

**FIG 1:**
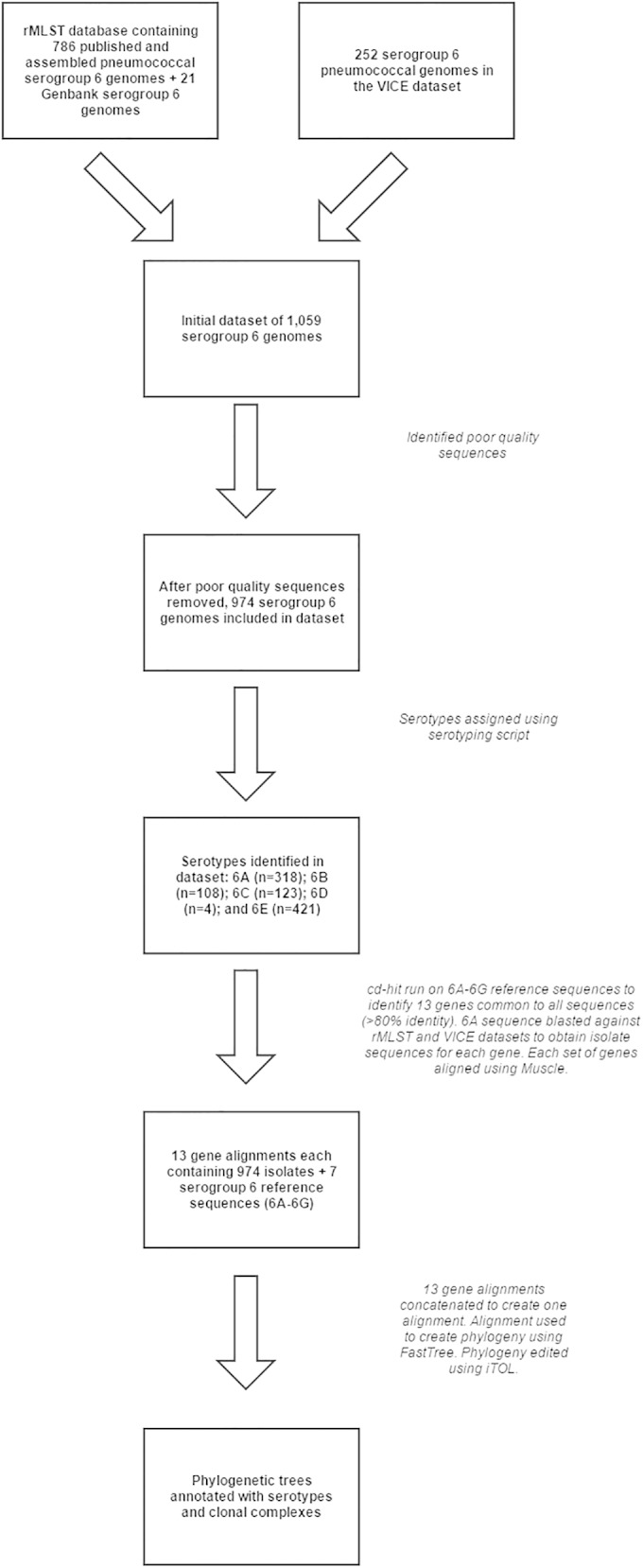
Flowchart outlining the compilation of the pneumococcal genome data set and corresponding *cps* locus sequences that were analyzed in this study.

A data set of 1,059 assembled serogroup 6 pneumococcal genome sequences was compiled by using previously published genome data sets (41–47), GenBank sequence data (http://www.ncbi.nlm.nih.gov/GenBank/), and unpublished genome data from an ongoing vaccine impact study in Iceland (Fig. 1; see Table S1 in the supplemental material). The vaccine impact study is collecting pneumococcal isolates from healthy children and from patients of all ages with pneumococcal disease and sequencing 3,100 isolates with the Illumina platform. Pre- and postvaccine pneumococci from 2009 to 2015 will be analyzed and compared, and a report on the complete data set will be published in due course. The genome data set included four serotype 6B pneumococcal reference strains from the PMEN collection (48) whose genome sequences were available (Table 1). The genomes from GenBank were downloaded directly, and all other genomes in the data set were downloaded as raw sequence reads from the European Nucleotide Archive (ENA), assembled with Velvet (49), and deposited in the rMLST database, which is powered by BIGSdb (50, 51). Corresponding metadata were manually acquired from the original publications and matched to the genome data. Genome sequence quality was assessed, and poor-quality sequences (e.g., those with gaps or non-full-length gene sequences in the *cps* locus) were removed, leaving 974 genomes for analysis. All of the genome assemblies analyzed in the present study, with the corresponding metadata, are available from the pneumococcal PubMLST website (http://pubmlst.org/spneumoniae/).

**TABLE 1:** Reference PMEN^*a*^ genomes and *cps* locus sequences used in this study

Reference	Other name	ST	Accession no.	Yr	Other information	PMID^*a*^
PMEN2	Spain^6B^-2	ST90	ATCC 700670; ENA	1988	Abscess in human patient, Madrid	8885390; 11427569
PMEN8	S. Africa^6B^-8	ST185	ATCC 700675; ENA	1990	Human blood culture, South Africa	9442492; 11427569
PMEN12	Finland^6B^-12	ST270	ATCC 700903; ENA	1987	Isolated in Finland	1398923; 11427569
PMEN17	Maryland^6B^-17	ST384	ATCC BAA342; ENA	1997	Invasive disease isolate, United States	10608770
*cps* locus of serotype:						
6A	34351 Rodrigues 6A/2	ST7188	CR931638	1952	Isolated in United States	16532061
6B	2616/39 6B/3	ST7199	CR931639	1939	Isolated in Denmark	16532061
6C	CHPA388		EF538714	1999–2002	Nasopharyngeal isolate, United States	17576753
6D	MNZ21	ST282	HM171374	2008	Isolated from nasopharynx of healthy child, South Korea	20929956
6E	HK02-14	ST90	Multiple (accession numbers for each sequenced gene, see reference)	2008	Isolated from sputum sample, Hong Kong	23824778
6F	MNZ1135 PS6657		KC832411	1992–2012	Clinical adult sample, Germany	23897812
6G	MNZ1136 PS16864		KC832410	1992–2012	Clinical adult sample, Germany	23897812

a PMEN, Pneumococcal Molecular Epidemiology Network; ST, sequence type as determined by multilocus sequence typing; PMID, PubMed identifier.

### Serotyping based on the *cps* locus sequence.

The *cps* locus sequences for each of the serogroup 6 references (serotypes 6A to 6G) were obtained from public databases (Table 1). The serotypes associated with each of the 974 serogroup 6 genomes were differentiated on the basis of the sequence of the *cps* locus genes with an in-house serotyping pipeline. Briefly, following an initial screening against the serotype reference sequences, serogroup 6 genomes were serotyped by identifying the following amino acid residues and/or alleles specific for each serotype: serotypes 6A and 6B, *wciP*_195_; serotypes 6C and 6D, presence of *wciN*β and *wzy*_117_; serotype 6E, *wzy*_220_ (for additional details, see Fig. S1 in the supplemental material or contact us).

### Analyses of *cps* locus sequences.

Thirteen *cps* locus genes were identified among all seven serogroup 6 reference sequences with cd-hit (52) by using a sequence identity threshold of >80%: *wzg*, *wzh*, *wzd*, *wze*, *wchA*, *wciO*, *wciP*, *wzy*, *wzx*, *rmlA*, *rmlC*, *rmlB*, and *rmlD* (see Fig. S2 in the supplemental material for phylogenetic trees for all 13 genes). Note that *wciN* was also present among all of the *cps* loci, but the α and β versions of *wciN* were highly divergent, as noted previously (29, 53). The serotype 6A reference sequence for each of the 13 genes was then BLASTed (54) against the study genome data set to extract the sequences of the 13 genes from each genome. Pairwise estimates of evolutionary distance (p-distance; number of nucleotide sites that differ between two sequences divided by the total number of nucleotides compared) were calculated for each of the 13 genes in the 974 genomes, stratified by serotype.

The extracted *cps* locus sequences were aligned gene by gene with Muscle (55) before being concatenated together to obtain a 12,271-bp *cps* locus alignment for each of the 974 genomes. The concatenated sequences were then input into FastTree2 to construct a *cps* locus phylogeny by using a nucleotide general time-reversible model (56). The resulting phylogeny was annotated with iTOL (57). The p-distances between the serogroup 6 *cps* locus reference sequences were calculated with MEGA5 (58). Input sequences were 13,416 bp in length and spanned the *cps* locus from the start of *wzg* through the end of *rmlD*, including intergenic regions but excluding *wciN*, *HG262* (present in the *cps* locus of serotypes 6A, 6F, and 6G only), and *HG263* (present in serotypes 6B and 6E only).

### Genome-wide assessment of sequence diversity.

The Genome Comparator module in BIGSdb was used to compare all 974 serogroup 6 genomes to the annotated reference genome 670-6B (NC_014498.1), also known as PMEN2 or Spain^6B^-2. The BLASTn parameters were set to ≥70% sequence identity and 100% sequence alignment length (50). The data were exported to an Excel spreadsheet that depicted the results of sequence comparisons of each annotated coding sequence (here referred to as a “gene” for simplicity) in the reference 670-6B genome to each of the 974 query genomes. Data were output on a gene-by-gene basis across the entire genome for every query genome. The 670-6B reference gene sequences were designated allele 1, and corresponding sequences in each query genome were designated X (not present), 1 (identical to the reference), N (sequence present but nonidentical to the reference, assigned allele numbers to indicate unique sequences), or T (sequence present but truncated). The exported Genome Comparator gene-by-gene data analysis for the full 2.24-Mb genome was read into R (http://www.r-project.org/) to create a pseudoheat map to compare gene presence or absence and sequence diversity across all 974 genomes.

The Genome Comparator analysis also revealed that 432 gene sequences were found in all 974 genomes in the full coding length; thus, for each of the genomes, these 432 genes were concatenated (292 kb in total) and FastTree2 and ClonalFrameML (59) were used to assemble a phylogenetic tree that represented all 974 genomes. The resulting phylogeny was annotated with iTOL. Multilocus sequence type (MLST) data were available for each genome, and the sequence types (STs) were clustered into clonal complexes (CCs) with Phyloviz (60) (see Table S1 in the supplemental material). The 432-gene phylogenetic tree was annotated with CC and serotype data for each genome.

### Serological analyses of serotype 6E pneumococci.

To evaluate functional antibody responses to serotype 6E, sera collected after primary vaccination from PCV7 and PCV13 recipients (*n* = 8) who participated in previous vaccine studies (61, 62) were selected. Sera were analyzed by an opsonophagocytosis assay (OPA; a titer of ≥1:8 is considered positive) (63) utilizing five different isolates of serotype 6E pneumococci (PMEN2 and PMEN8 plus three recent Icelandic isolates). To assess the contribution of serotype-specific antibody in mediating the killing of cross-reactive antigens, sera were retested by OPA after the sera were adsorbed with purified serotype 6A, 6B (American Type Culture Collection, Manassas, VA), or 6C capsular polysaccharide (Statens Serum Institut, Copenhagen, Denmark) one at a time and reported as the titer where 50% of bacteria were killed.

## RESULTS

### Epidemiology of serogroup 6 pneumococci.

The study genome data set represented a diverse set of 974 serogroup 6 pneumococci recovered in 16 different countries across five continents between 1972 and 2014 (Table 2). Of the pneumococci recovered from individuals spanning a wide range of ages, 69% (*n* = 675) were from colonized individuals and 27% (*n* = 267) were from individuals with disease. A total of 78% (*n* = 760) of the pneumococci were collected prior to any PCV introduction in the country of origin. Antibiogram data demonstrated a range of antimicrobial-susceptible and -resistant isolates among the serotypes, although susceptibility data were missing for many of the pneumococcal genomes. For the full list of genomes and associated metadata, see Table S1 in the supplemental material. A majority of the pneumococci in this study were originally serotyped by the Quellung and/or latex agglutination methods. See Fig. S3 in the supplemental material for a comparison of the serotype distributions among all 974 genomes using the original serotyping data versus sequence-based serotyping (serotype deduced on the basis of the *cps* locus sequence).

**TABLE 2:** Epidemiological characteristics of individual serotypes^*a*^ within 974 serogroup 6 pneumococcal genomes

Characteristic	6A	6B	6C	6D	6E	Hybrid	Total
No. (%) of isolates	318 (33)	108 (11)	115 (12)	4 (0.4)	421 (43)	8	974
Yr of isolation	1972–2013	2001–2013	2001–2014	2004	1981–2013	2008–2010	1972–2014
No. of isolates from:							
Thailand	125	0	61	4	202	8	400
Iceland	118	100	14	0	130	0	362
United States	53	8	40	0	20	0	121
South Africa	19	0	0	0	10	0	29
Portugal	0	0	0	0	13	0	13
Germany	0	0	0	0	10	0	10
South Korea	0	0	0	0	8	0	8
Spain	0	0	0	0	7	0	7
China	0	0	0	0	6	0	6
Peru	0	0	0	0	6	0	6
Turkey	2	0	0	0	1	0	3
France	0	0	0	0	2	0	2
Finland	0	0	0	0	1	0	1
Oman	0	0	0	0	1	0	1
Papua New Guinea	1	0	0	0	0	0	1
Unknown	0	0	0	0	4	0	4
No. of isolates recovered from:							
Carriage	247	80	107	4	229	8	675
Disease	67	28	8	0	164	0	267
Unknown	4	0	0	0	28	0	32
Patient age (yr)^*b*^	<0.5–87	0.5–83	<0.5–82	unknown	<0.5–87	unknown	<0.5–87
No. of isolates recovered:^*c*^							
Before PCV	227	73	69	4	379	8	760
After PCV	89	35	46		35		205
Unknown	2				7		9
MIC (μg/ml)^*d*^ of:							
Penicillin	<0.03–16	<0.03–0.06	<0.03–1	S^*d*^	<0.03–4	0.06–1	<0.03–16
Erythromycin	<0.03–16	<0.03–0.06	<0.03–2		<0.03–256		<0.03–256
Tetracycline	≤0.5 to >4	≤0.5–0.25	≤0.5–0.25		≤0.5–64		≤0.5–64
Chloramphenicol	2	2	2–4		2 to >8		2 to >8

a Serotypes were determined from the nucleotide sequence of the *cps* locus. No pneumococci of serotype 6F or 6G were identified in the study genome data set. The hybrid serotype is genetically a combination of the serotype 6C and 6E *cps* locus sequences (see Results).

b Age data were missing for 625 genomes, but available data indicated that isolates of serotypes 6A, 6B, 6C, and 6E were recovered from both children and adults.

c Vaccine status refers to whether any pneumococcal conjugate vaccine (PCV) was being used in the country of origin at the time of pneumococcus isolation.

d Susceptibility data were missing for many genomes, but the ranges of available data are given here. S, susceptible. See Table S1 in the supplemental material for more details.

Sequence-based analysis of the data set revealed that 421 of the 974 pneumococci were serotype 6E. They were recovered from individuals between the ages of 6 months and 87 years residing in 15 different countries in Europe, North America, South America, Africa, and Asia (Table 2). The serotype 6E pneumococci were isolated from 1981 onward, and 90% were isolated prior to the use of any PCVs. Serotype 6E isolates were recovered from both healthy young children and individuals of all ages with pneumococcal disease. The diseases caused by serotype 6E pneumococci spanned the range of typical pneumococcal diseases, i.e., otitis media, sinusitis, empyema, pneumonia, bacteremia, and meningitis (see Table S1 in the supplemental material).

One-third (*n* = 318) of the genomes were serotype 6A pneumococci recovered between 1972 and 2013 from patients of all ages residing in six different countries. The majority of the serotype 6A pneumococci were recovered from healthy children, although 67 isolates from patients with invasive and noninvasive diseases were also included (Table 2; see Table S1 in the supplemental material). Pneumococci with a serotype 6C *cps* locus sequence made up 12% (*n* = 115) of the data set and were recovered predominantly from healthy children in Thailand, Iceland, and the United States since 2001.

Eleven percent (*n* = 108) of the pneumococci possessed a serotype 6B *cps* sequence. Serotype 6B pneumococci were isolated between 2001 and 2013 from both carriage and disease (otitis media, pneumonia, and bacteremia), from patients of a wide range of ages. Four serotype 6D pneumococci from Thailand were identified among the genomes, but no serotype 6F or 6G pneumococci were identified. Eight carriage pneumococci from Thailand with a hybrid serotype 6C/6E *cps* locus were also identified, and these are discussed in more detail below.

### Serotype-specific prevalence estimates.

The study genome data set was diverse and compiled from several different genome collections; thus, estimates of serotype-specific prevalence based on the entire data set may not be representative of the global pneumococcal population. However, the carriage data sets from the Maela refugee camp in Thailand and from Massachusetts could reliably be used to assess the prevalence of serotypes within specific geographic locations during a specified time period.

The Maela genome data set was made up of 3,085 pneumococci collected from infants and mothers living in a rural refugee camp in Thailand from 2007 to 2010 (41, 64). PCVs had not been used in this setting prior to or during the study period. Three hundred ninety-eight serogroup 6 pneumococcal genomes were identified, of which 50% (*n* = 200) were serotype 6E, 31% (*n* = 125) were serotype 6A, 15% (*n* = 61) were serotype 6C, 1% (*n* = 4) were serotype 6D, and 2% (*n* = 8) were the hybrid 6C/6E serotype (see Table S1 in the supplemental material). No pneumococci with a serotype 6B *cps* locus sequence were identified.

In contrast, 616 pneumococcal genomes were collected from healthy young children in Boston, MA, during three time periods (2001, 2004, and 2007) after the implementation of PCV7 (42). Ninety-seven serogroup 6 pneumococci were identified, of which 47% (*n* = 46) were serotype 6A, 35% (*n* = 34) were serotype 6C, 9% (*n* = 9) were serotype 6E, and 8% (*n* = 8) were serotype 6B (see Table S1 in the supplemental material).

### Serotypes among PMEN clones.

Genome sequences of four PMEN reference strains were included in this study, PMEN2 (Spain^6B^-2), PMEN8 (S. Africa^6B^-8), PMEN12 (Finland^6B^-12), and PMEN17 (Maryland^6B^-17), as shown in Table 1 (48; http://pubmlst.org/spneumoniae/). All four were previously identified as serotype 6B on the basis of the Quellung reaction, but all possess a serotype 6E *cps* locus sequence. Note that the *cps* locus sequence of Poland^6B^-20 was not yet available and the genome sequence of Greece^6B^-22 was incomplete for some of the *cps* locus genes and thus could not be analyzed in this study. Moreover, one data set included in this study was compiled specifically to study the PMEN2 lineage (43), which is a multidrug-resistant lineage of pneumococci detected in many countries around the world but was originally identified in Iceland and Spain in the 1980s (http://pubmlst.org/spneumoniae/). One hundred eighty-nine pneumococcal genomes were sequenced in the original PMEN2 study. One hundred seventy-two of these had complete *cps* locus sequences and were thus included in the present study, and all possessed a serotype 6E *cps* locus sequence.

### Genetics and phylogeny of the *cps* locus.

**FIG 2:**
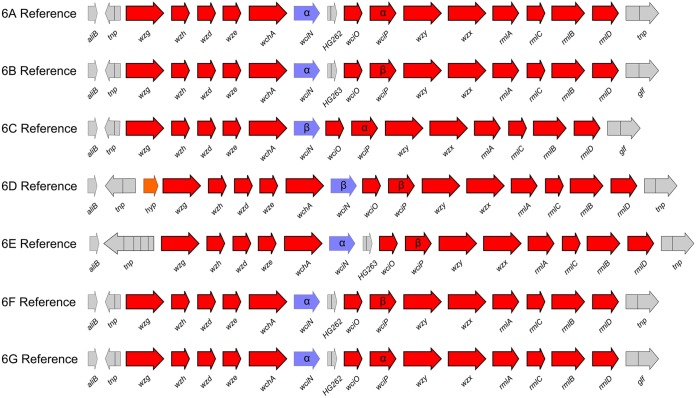
Organization of the *cps* locus for each of the seven serogroup 6 reference sequences (Table 1 contains the accession numbers). Red arrows are the 13 genes common to all serogroup 6 *cps* loci at >80% sequence similarity, *wciN* is blue, and the α or β allele is indicated, transposons and other pseudogenes are gray, and a hypothetical gene (*hyp*) is orange. The α and β versions of *wciP* are also indicated.

Thirteen *cps* locus genes were common to all 974 serogroup 6 pneumococci at a similarity threshold of >80% (Fig. 2), and synteny was preserved among all of the common genes. Pairwise distances (p-distances) of nucleotide variation between the *cps* loci of the seven reference strains were calculated, and notably, serotype 6E was 6.7% divergent (p-distance range, 0.065 to 0.068) from all of the other serotypes, whereas the divergences between all of the other serotypes were 0.4 to 1.7% (Table 3). The p-distances were also calculated individually for each of the 13 common genes by using the entire 974-genome data set stratified by serotype, and the median p-distance value was used to provide a simple summary statistic of gene-specific sequence diversity within each serotype (Table 3). Most notably, the serotype 6B, 6D, and 6E *cps* gene sequences were highly conserved within each serotype (median p-distance per gene = 0), whereas nearly all of the *cps* locus genes of the serotype 6A and 6C isolates varied to some extent, with *wzg*, *rmlA*, and *rmlB* being the most diverse (in addition to *wciN*), as noted in a previous study (37).

**TABLE 3:** Estimates of pairwise evolutionary distances between serogroup 6 *cps* locus sequences and among individual genes within the *cps* locus of each serotype

*cps* locus^*a*^ or gene^*b*^	Evolutionary distance from indicated serotype^*c*^						
	6A	6B	6C	6D	6E	6F	6G
6A	—	0.008	0.016	0.011	0.068	0.009	0.010
6B	0.008	—	0.011	0.016	0.065	0.008	0.004
6C	0.016	0.011	—	0.012	0.066	0.010	0.013
6D	0.011	0.016	0.012	—	0.068	0.012	0.017
6E	0.068	0.065	0.066	0.068	—	0.067	0.067
6F	0.009	0.008	0.010	0.012	0.067	—	0.009
6G	0.010	0.004	0.013	0.017	0.067	0.009	—
*wzg*	0.013	0	0	0	0	—	—
*wzh*	0.008	0	0	0	0	—	—
*wzd*	0.001	0	0.001	0	0	—	—
*wze*	0.004	0	0.009	0	0	—	—
*wchA*	0.004	0	0.007	0	0.001	—	—
*wciN*α	0.001	0	—	—	0	—	—
*wciN*β	—	—	0.002	0	—	—	—
*wciO*	0	0	0.006	0	0	—	—
*wciP*	0	0	0.002	0	0	—	—
*wzy*	0.001	0	0.004	0	0	—	—
*wzx*	0.001	0	0.001	0	0	—	—
*rmlA*	0.046	0	0.062	0	0	—	—
*rmlC*	0.005	0	0.002	0	0	—	—
*rmlB*	0.016	0	0.014	0	0	—	—
*rmlD*	0.005	0	0.006	0	0	—	—

a Pairwise comparisons were made between the serogroup 6 references by using 13,416-bp *cps* locus sequences, which spanned the *cps* locus from the start of *wzg* through the end of *rmlD* but excluded *wciN*, *HG262*, and *HG263* from the analysis (see Materials and Methods and Fig. 2).

b Median pairwise distances for each *cps* locus gene were estimated by using the entire 974-pneumococcal-genome data set but stratified by serotype. No serotype 6F or 6G pneumococci were identified among the study genomes.

c —, data not available.

**FIG 3:**
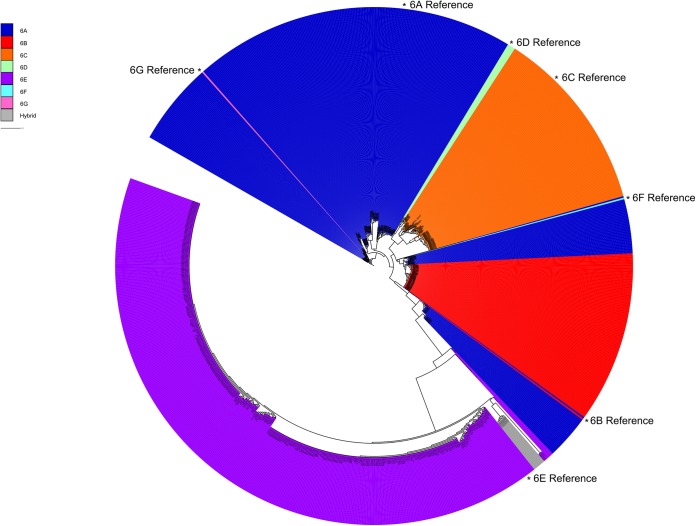
Phylogenetic tree depicting the relationships between the concatenated sequences of 13 common *cps* locus genes (12.3 kb) among 974 serogroup 6 pneumococcal genomes. Serotypes are colored according to the legend in the top left corner.

A phylogenetic tree was constructed with concatenated sequences of the 13 common *cps* locus genes for each of the 974 genomes, and major serotype clusters were clearly delineated (Fig. 3). There were three major serotype 6A clusters and one cluster each for serotypes 6B, 6C, and 6D. No serotype 6F or 6G pneumococci were identified, but the serotype 6F and 6G reference sequences were within serotype 6A clusters. This was consistent with the initial report describing these new serotypes as being nearly identical to the serotype 6A *cps* sequence (10). The serotype 6E pneumococci clustered in one group with minor within-cluster genetic variation, apart from a few pneumococci from South Africa in 1984 and 1985 and Massachusetts in 2004 (long purple branches) that had switched serotypes and are discussed below, and three Thai pneumococci at the tips of very short branches that possess predominantly the serotype 6E *cps* locus sequence but also have sequence regions that match the serotype 6C or 6D sequence.

**FIG 4:**
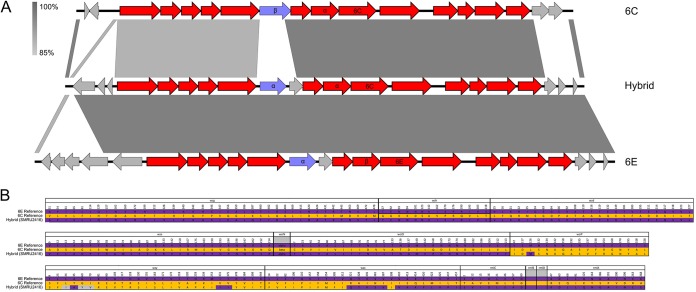
Illustration of the genetic organization, nucleotide similarity, and variable amino acids of the hybrid serotype 6C/6E *cps* locus sequence. (A) Comparison of the hybrid *cps* locus sequence to the reference serotype 6C and 6E sequences. The results of pairwise BLAST nucleotide sequence comparisons are shown; darker gray indicates greater conservation between the pair of sequences. (B) Variable amino acid residues identified among the serotype 6C and 6E and hybrid sequences. The position of each variable residue in an alignment of conceptually translated amino acid sequences is indicated by the number above the residue. The residues associated with serotype 6E and 6C are purple and orange, respectively, and those not found in either serotype 6E or serotype 6C are gray. Note that the *wciN* allele is simply indicated as α or β and that *rmlB* and *rmlD* were identical across all three sequences.

There was also a small cluster (colored gray) of eight pneumococci collected in Thailand from 2008 to 2010. This small cluster represented a hybrid serotype 6C/6E *cps* locus, with sequence differences as shown in Fig. 4. The hybrid failed to be classified properly as either serotype 6C or serotype 6E in the serotyping pipeline because it possessed *wciN*α like serotype 6E but the *wzy*-encoded amino acid sequence that differentiates serotype 6C (Fig. 4A). The reason for this serotyping failure was clearly apparent when the sequences for each of the *cps* locus genes was examined (Fig. 4B). The hybrid predominantly matches the serotype 6E sequence but contains a region of mainly serotype 6C-like sequence from *wciP* through roughly the first half of *wzx*.

### Molecular epidemiology of serogroup 6 pneumococci.

**FIG 5:**
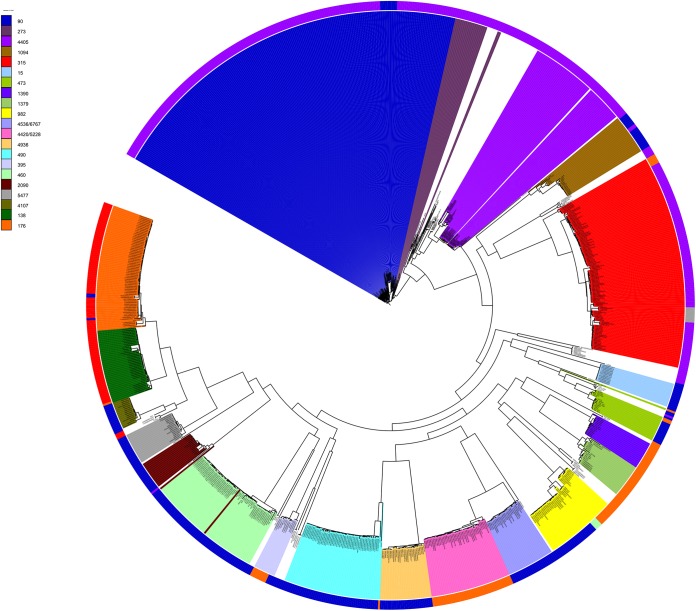
Phylogenetic tree describing genome-wide relationships among serogroup 6 pneumococci. The tree was constructed with the concatenated sequences of 432 full-length coding loci found in all 974 genomes and annotated with CC designations and serotypes. CCs with ≥12 isolates are colored as shown in the key at the upper left. The outer ring indicates the serotype by color as follows: 6A, blue; 6B, red; 6C, orange; 6D, light green; 6E, purple; 6F, light blue; 6G, pink.

A phylogenetic tree was constructed on the basis of 432 concatenated gene sequences (292 kb) present in full coding length in all 974 pneumococci (Fig. 5). The tree was annotated by using CC and serotype data, which clearly defined the major CCs (those with ≥12 members) within the data set. The serotype data, represented by the outer colored ring, demonstrated which serotypes were associated with each CC and where serotype switching had occurred within CCs.

Serotype 6E pneumococci were associated with 20 CCs (43 sequence types [STs]) in the study data set, although 89% of the serotype 6E pneumococci were members of one of four lineages, CC90 (*n* = 203); CC315 (*n* = 117); CC4405 (*n* = 55); and CC273 (*n* = 20), as shown in Fig. 5 (also see Table S1 in the supplemental material). CC90, CC315, and CC273 are multidrug-resistant lineages associated with PMEN2, PMEN20 (Poland^6B^-20), and PMEN22 (Greece^6B^-22), respectively (see Table S1 in the supplemental material; http://pubmlst.org/spneumoniae/). All eight hybrid serotype 6C/6E genomes were ST315. Notably, pneumococci of these four serotype 6E lineages have been detected in at least 32 different countries on six continents, as detailed in the PubMLST database. However, the majority of the isolates of these four lineages in PubMLST were submitted as serotype 6B (including PMEN20 and PMEN22); these should be considered putative serotype 6E rather than serotype 6B, and thus, the PubMLST database expands the wide distribution of serotype 6E pneumococci.

Serotype 6A pneumococci were members of 24 different CCs (45 STs) in the study data set, of which 11 CCs captured 90% of the serotype 6A population (see Table S1 in the supplemental material). Serotype 6C pneumococci were associated with 12 CCs, 5 of which defined 83% of the serotype 6C genomes. All but three strains of serotype 6B pneumococci were members of either CC176 (*n* = 66) or CC138 (*n* = 39), and all four serotype 6D genomes were ST4407.

### Serotype switching among serogroup 6 pneumococci.

**FIG 6:**
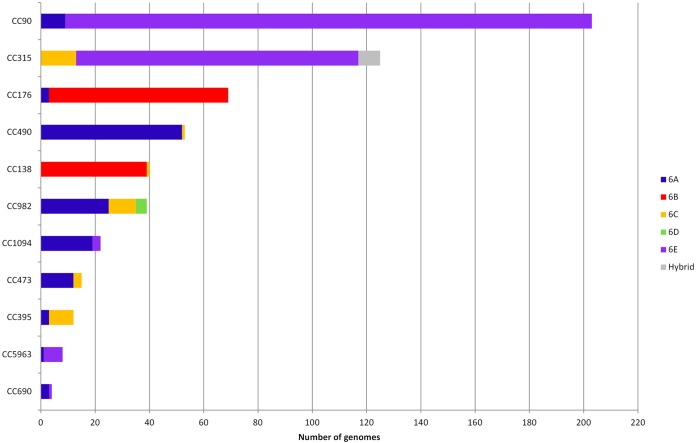
CCs identified among the 974 pneumococcal genomes that were not of a single serotype.

There was clear evidence of serotype switching (horizontal genetic exchange of all or part of the *cps* locus sequence, conferring a change in serotype) within 11 CCs, since pneumococci within the CC were not exclusively defined by a single serotype (Fig. 5 and 6). Notably, CC315 was represented by three serotypes: serotypes 6E and 6C and the serotype 6C/6E hybrid. CC90 was defined predominantly by serotype 6E pneumococci, except for nine genomes from the Maela refugee camp that were ST5127 (a double-locus variant of ST90) and had a serotype 6A *cps* locus. CC1094 was a major South African lineage of serotype 6A, although three genomes isolated in the mid-1980s were serotype 6E.

The *cps* locus sequences of all 44 genomes associated with putative serotype switches were manually inspected and confirmed. All serotype switches were clearly evident from the sequence, either by an exchange of the entire *cps* locus or by mosaic patterns of DNA sequence fragments indicative of recombination events within the *cps* locus (see Fig. S4 in the supplemental material) (17, 18, 22–25). No other hybrid *cps* loci were identified apart from the serotype 6C/6E hybrid described above.

### Genome-wide diversity among serotypes.

**FIG 7:**
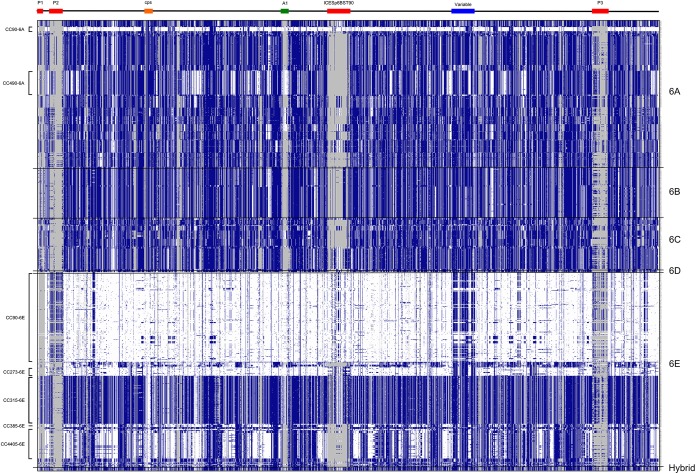
Visual representation of the Genome Comparator output for all 974 genomes as a pseudoheat map. Each genome is depicted horizontally and in the gene order defined by the reference PMEN2 genome sequence, which has 2,352 coding sequences (genes). Colors indicate the gene-by-gene presence or absence and sequence similarity of each query genome compared to the PMEN2 reference as follows: gray, the gene is not present in the query genome; white, the gene is present in the query genome and has a sequence identical to that of the reference genome; blue, the gene is present in the query sequence, but the sequence is not identical to that of the reference. Several regions of the PMEN2 reference genome are highlighted as follows: P1, phage remnant; P2, 11865-like phage; P3, 2167-like phage; cps, capsular locus; A1, ATP-synthase operon; ICESp6BST90, ICE element; Variable, variable region of the gene sequences in CC90-6E.

Finally, all 974 genomes were compared to the PMEN2 genome reference with the Genome Comparator module of BIGSdb to investigate the presence or absence and diversity of 2,352 genes across each genome. These data were depicted as a pseudoheat map, ordered by serotype and CC (Fig. 7). A number of observations were immediately apparent. As expected, the genomes of CC90^6E^ (CC^serotype^) pneumococci were very similar to the PMEN2 reference (ST90^6E^) genome used for comparison (largest white horizontal band). However, they were not identical—the bacteriophage sequences in the PMEN2 reference were either not present or of a different sequence in the CC90^6E^ study pneumococci, and there was a region of variable genes (blue) in the latter half of the genome in addition to several smaller variable regions in some genomes within CC90^6E^. The serotype switch CC90^6A^ pneumococci were highly similar across the genome to the CC90^6E^ pneumococci, although CC90^6A^ genomes also did not have the PMEN2-like bacteriophages and smaller variable regions were identified. The STs within CC273^6E^, CC4405^6E^, and CC490^6A^ had some MLST alleles (5, 1-4, and 1, respectively) in common with the STs in CC90, but all possessed genes across the genome that matched CC90^6E^ identically (genes colored white). In contrast, across the genome, the CC315^6E^ genes were mainly of different alleles (i.e., mainly genes colored blue). Future studies will use these genome-wide data to investigate whether specific genotypic differences among serogroup 6 lineages relate to phenotypic differences between lineages and/or serotypes.

### Vaccine-induced inhibition of serotype 6E.

Finally, a key question was whether or not PCVs would provide immunological protection against serotype 6E pneumococci. Stored sera from infants in the United Kingdom who had previously been vaccinated with either PCV7 or PCV13 were available and used to test for killing of serotype 6E pneumococci. Five strains were tested, and the results are shown in Table 4. Antibodies induced by both PCV7 and PCV13 mediated the killing of the five strains tested. Removal of antibodies specific for serotype 6B polysaccharide abolished the killing completely in most of the sera tested. Removal of serotype 6A antibodies varied by serum analyzed but in general was similar, irrespective of the type of vaccine used (6B alone in PCV7 or both 6B and 6A in PCV13), with only partial inhibition of killing demonstrated, findings consistent with those of a previous study of serotype 6A inhibition of serotype 6B killing (31). Anti-serotype 6C antibody removal also varied depending on the serum used but in general had little effect on killing.

**TABLE 4:** Results of serological assays using pediatric sera collected after primary PCV7 and PCV13 immunization to inhibit serotype 6E pneumococci

Serotype 6E strain, vaccine, and sample	Titer without competitor	Titer with serotype 6A PnPs^*a*^	% Inhibition	Titer with serotype 6B PnPs^*b*^	% Inhibition	Titer with serotype 6C PnPs	% Inhibition
PMEN2							
PCV7							
209(6)	266	NA^*c*^	NA	<8	98.5	298	−12.0
214(6)	165	117	29.1	<8	97.6	144	12.7
190(6)	3,462	3,738	−8.0	<8	99.9	2,999	13.4
154(6)	2,005	1,361	32.1	<8	99.8	1,397	30.3
042(7)	213	70	67.1	<8	98.1	258	−21.1
PCV13							
010A	766	708	7.6	<8	99.5	684	10.7
208A	261	147	43.7	<8	98.5	193	26.1
226A	1,995	1,684	15.6	<8	99.8	1,337	33.0
240A	2,030	1,760	13.3	496	75.6	1,835	9.6
243A	3,069	433	85.9	NA	NA	2,473	19.4
PMEN8							
PCV7							
209(6)	246	NA	NA	<8	98.4	237	3.7
214(6)	235	221	6.0	<8	98.3	237	−0.9
190(6)	2,382	3,811	−60.0	<8	99.8	2,692	−13.0
154(6)	2,151	1,755	18.4	<8	99.8	1,894	11.9
042(7)	477	90	81.1	<8	99.2	585	−22.6
PCV13							
010A	484	536	−10.7	<8	99.2	456	5.8
208A	111	72	35.1	<8	96.4	75	32.4
226A	1,677	1,446	13.8	<8	99.8	1,245	25.8
240A	1,979	1,803	8.9	533	73.1	2,153	−8.8
243A	2,769	328	88.2	<8	99.9	2,117	23.5
VICE^*d*^ 0629							
PCV7							
209(6)	NA	NA	NA	NA	NA	NA	NA
214(6)	180	54	70.0	<8	97.8	112	37.8
190(6)	2,024	1,877	7.3	<8	99.8	1,858	8.2
154(6)	923	790	14.4	<8	99.6	703	23.8
042(7)	<8	<8	0.0	<8	0.0	<8	0.0
PCV13							
010A	420	199	52.6	<8	99.0	193	54.0
208A	NA	NA	NA	NA	NA	NA	NA
226A	681	509	25.3	<8	99.4	566	16.9
240A	1,316	1,011	23.2	249	81.1	977	25.8
243A	1,419	117	91.8	<8	99.7	1,109	21.8
VICE 1004							
PCV7							
209(6)	198	NA	NA	<8	98.0	194	2.0
214(6)	359	334	7.0	<8	98.9	333	7.2
190(6)	2,514	2,424	3.6	<8	99.8	2,238	11.0
154(6)	863	1,141	−32.2	<8	99.5	762	11.7
042(7)	233	76	67.4	<8	98.3	239	−2.6
PCV13							
010A	421	488	−15.9	<8	99.0	461	−9.5
208A	233	100	57.1	<8	98.3	148	36.5
226A	2,626	241	90.8	NA	NA	1,855	29.4
240A	1,791	1,394	22.2	<8	99.8	1,257	29.8
243A	2,099	1,701	19.0	371	82.3	1,739	17.2
VICE 1150a							
PCV7							
209(6)	230	192	16.5	<8	98.3	247	−7.4
214(6)	367	336	8.4	<8	98.9	416	−13.4
190(6)	2,426	2,418	0.3	<8	99.8	2,368	2.4
154(6)	1,259	1,066	15.3	<8	99.7	1,470	−16.8
042(7)	500	161	67.8	<8	99.2	390	22.0
PCV13							
010A	540	293	45.7	<8	99.3	304	43.7
208A	145	106	26.9	<8	97.2	92	36.6
226A	1,674	1,173	29.9	<8	99.8	879	47.5
240A	1,602	931	41.9	238	85.1	944	41.1
243A	2,324	301	87.0	<8	99.8	1,849	20.4

a PnPs, pneumococcal polysaccharides (tested at 1 μg/ml).

b A titer of 4 was used to calculate percent inhibition for results of <8.

c NA, not available because of technical failure.

d VICE refers to a pneumococcal strain from the ongoing vaccine impact study in Iceland (see Materials and Methods).

## DISCUSSION

This is the first in-depth large-scale interrogation of the genomic epidemiology of serogroup 6 strains, and it illustrates that serotype 6E pneumococci have been circulating for at least 33 years, preceding PCV introduction by nearly 2 decades. Our study revealed that 43% of the genome collection (previously thought to contain predominantly serotypes 6A, 6B, and 6C) in fact represented serotype 6E pneumococci of several major genetic lineages, three of which were multidrug-resistant PMEN lineages. They were distributed across 15 countries and five continents, and the pneumococcal PubMLST database provides evidence of an even wider geographical distribution. Serotype 6E pneumococci caused a range of diseases among all age groups but were also frequently recovered from healthy young children. We identified several major genetic clusters of serotype 6A *cps* locus sequences, discovered a new hybrid 6C/6E serotype, and revealed many examples of serotype switching involving serotypes 6A, 6B, 6C, 6D, and 6E. Importantly, serological assays demonstrated that vaccine-induced serotype 6B antibodies were able to mediate the killing of serotype 6E pneumococci.

For several decades, the existence of serotype 6E pneumococci was obscured because they cross-react to the serotype 6B antisera used in the Quellung reaction, and it was only by inspection of the *cps* locus sequences that the existence of serotype 6E was realized. Initially, serotype 6E was recognized by several research groups analyzing key regions of gene sequences within small collections of isolates, and now the high prevalence and worldwide distribution of serotype 6E has been revealed unequivocally here by the interrogation of a global and historical collection of genome sequences. Basically, the majority of what for many years were thought to be serotype 6B isolates were in fact serotype 6E pneumococci. “True” serotype 6B pneumococci were also identified in our study, but they were mainly of two genetic lineages, CC138 and CC176, both of which have also been detected in many countries around the world (http://pubmlst.org/spneumoniae/).

Our study revealed the sequence diversity among serogroup 6 *cps* loci, and of particular note was the finding of three major serotype 6A *cps* locus sequence clusters. Do the sequence-based changes in the serotype 6A *cps* locus result in changes to the polysaccharide, and if so, do PCV-induced serotype 6A antibodies differentially protect against alternative versions of serotype 6A polysaccharide? This warrants further investigation. Moreover, in this study, we discovered the serotype 6C/6E hybrid, which is sufficiently divergent to presumptively consider it yet another serotype, as well as evidence of many distinct serotype switches among the genetic lineages. Yet it is important to recognize that serotype switching and the creation of *cps* locus genetic variants appear to be normal biological processes among pneumococci and are not a direct consequence of vaccine use (25). However, vaccine-induced immune pressure does alter the pneumococcal population structure, which can select for the emergence of new genetic variants. The earliest reported evidence of vaccine escape pneumococci was the result of such a scenario (17–19). PCVs perturb the pneumococcal population with unpredictable consequences for those serotypes not targeted by the vaccines; therefore, the importance of genomics and molecular epidemiology in any pneumococcal surveillance program cannot be underestimated.

A detailed comparison of serogroup 6 polysaccharide biochemistry should be investigated as a matter of priority, to understand the biochemical structure of the polysaccharides in the context of the observed serotype-specific epidemiology and killing mediated by serotype 6B antibodies. It may be that the structures of the serotype 6B and 6E polysaccharides are similar enough to explain the inhibition of serotype 6E pneumococci by PCV-induced serotype 6B antibodies, even though at the sequence level the *cps* loci are very different. However, Oliver et al. recently reported a detailed investigation of new serotypes 6F and 6G and experimentally showed that single changes in the amino acid sequence encoded by *wciN*α resulted in changes in the repeating units of the capsular polysaccharides, with concomitant changes in the serological profiles. *wciN*α and *wciN*β are highly divergent versions of *wciN*; serotypes 6A, 6B, 6E, 6F, and 6G possess *wciN*α (encoding a galactosyltransferase), while serotypes 6C and 6D have the *wciN*β allele (encoding a glucosyltransferase). The authors concluded that small changes in the sequence not only resulted in new capsular types, but they also posited that these changes could confer immunological changes in the human host response (10).

The majority of these serotype 6E study isolates were collected before PCV implementation, and our study showed that PCV7- and PCV13-induced antibodies to serotype 6B were protective. Therefore, the overall prevalence of serotype 6E should be significantly reduced after PCV implementation, and PCV vaccine impact studies in many countries have demonstrated a significant reduction in the prevalence of serotype 6B and near elimination of serotype 6B carriage (5, 6). This suggests the possibility that (i) PCV7 and PCV13, which contain serotype 6B polysaccharides, inhibit serotype 6B and sufficiently cross-protect against serotype 6E at the population level or (ii) the “serotype 6B” polysaccharides in the vaccines are actually serotype 6E polysaccharides. We have been unable to identify which strain(s) was specifically used to produce the serotype 6B polysaccharides used in the PCVs, in order to confirm the serotype on the basis of the *cps* locus sequence.

Whether or not current PCVs are, in fact, serotype 6E vaccines remains an open and important question. The overwhelming success of PCVs in reducing serotype 6B (6E) disease suggests that perhaps the question is purely an academic one; however, it would be relevant in cases of vaccine failure where there may be discordance between the vaccine serotype and the serotypes of pneumococci associated with vaccine failure. One clue to a mismatch might be if pneumococci responsible for serotype 6B vaccine failures were of the ST138 or ST176 lineage, as these appear to be the predominant (true) serotype 6B lineages that circulate worldwide. Country-specific estimates of the prevalence of serotype 6E before and after PCV implementation will be essential to assessing whether PCVs are protective against serotype 6E at the population level.

Genomics has revolutionized microbiological research, and our study reinforces just how influential the change has been and will continue to be. It is now relatively simple and cost-effective to use next-generation sequencing to obtain a (nearly) complete bacterial genome sequence, and the early challenges of contig assembly, the availability of databases in which to store and query genome sequences, and the development of tools for the analysis of large-scale databases are being overcome. There are currently >10,000 pneumococcal genome sequences available in public databases, and other genome sequencing projects are under way. Challenges remain, including making published genome assemblies widely accessible to all users, but the genomics field is moving apace.

## Supplementary Material

Supplemental material

## References

[1] Wardlaw T, White Johansson E, Hodge M 2006 Pneumonia, the forgotten killer of children. United Nations Children's Fund/World Health Organization, Geneva, Switzerland http://apps.who.int/nuvi/integration/Pneumonia_The_Forgotten_Killer_of_Children.pdf.

[2] Wardlaw T 2014 Committing to child survival: a promise renewed. United Nations Children's Fund, New York, NY http://www.apromiserenewed.org/APR_2014_web_15Sept14.pdf.

[3] O'Brien KL, Wolfson LJ, Watt JP, Henkle E, Deloria-Knoll M, McCall N, Lee E, Mulholland K, Levine OS, Cherian T 2009 Burden of disease caused by Streptococcus pneumoniae in children younger than 5 years: global estimates. Lancet 374:893–902. doi:10.1016/S0140-6736(09)61204-6. 19748398

[4] Drijkoningen JJ, Rohde GG 2014 Pneumococcal infection in adults: burden of disease. Clin Microbiol Infect 20(Suppl 5):45–51. doi:10.1111/1469-0691.12461. 24313448

[5] World Health Organization. 2012 Pneumococcal vaccines WHO position paper—2012. Wkly Epidemiol Rec 87:129–144. http://www.who.int/wer/2012/wer8714.pdf. 24340399

[6] Fitzwater SP, Chandran A, Santosham M, Johnson HL 2012 The worldwide impact of the seven-valent pneumococcal conjugate vaccine. Pediatr Infect Dis J 31:501–508. 22327872 10.1097/INF.0b013e31824de9f6

[7] Black S, Shinefield H, Fireman B, Lewis E, Ray P, Hansen JR, Elvin L, Ensor KM, Hackell J, Siber G, Malinoski F, Madore D, Chang I, Kohberger R, Watson W, Austrian R, Edwards K 2000 Efficacy, safety and immunogenicity of heptavalent pneumococcal conjugate vaccine in children. Northern California Kaiser Permanente Vaccine Study Center Group. Pediatr Infect Dis J 19:187–195. 10749457 10.1097/00006454-200003000-00003

[8] Yeh SH, Gurtman A, Hurley DC, Block SL, Schwartz RH, Patterson S, Jansen KU, Love J, Gruber WC, Emini EA, Scott DA 2010 Immunogenicity and safety of 13-valent pneumococcal conjugate vaccine in infants and toddlers. Pediatrics 126:e493-505. doi:10.1542/peds.2009-3027. 20732948

[9] Silfverdal SA, Hogh B, Bergsaker MR, Skerlikova H, Lommel P, Borys D, Schuerman L 2009 Immunogenicity of a 2-dose priming and booster vaccination with the 10-valent pneumococcal nontypeable Haemophilus influenzae protein D conjugate vaccine. Pediatr Infect Dis J 28:e276-82. doi:10.1097/INF.0b013e3181b48ca3. 20118683

[10] Oliver MB, van der Linden MPG, Küntzel SA, Saad JS, Nahm MH 2013 Discovery of Streptococcus pneumoniae serotype 6 variants with glycosyltransferases synthesizing two differing repeating units. J Biol Chem 288:25976–25985. doi:10.1074/jbc.M113.480152. 23897812 PMC3764802

[11] Calix JJ, Nahm MH 2010 A new pneumococcal serotype, 11E, has a variably inactivated *wcjE* gene. J Infect Dis 202:29–38. doi:10.1086/653123. 20507232 PMC2880655

[12] Calix JJ, Porambo RJ, Brady AM, Larson TR, Yother J, Abeygunwardana C, Nahm MH 2012 Biochemical, genetic, and serological characterization of two capsule subtypes among Streptococcus pneumoniae serotype 20 strains: discovery of a new pneumococcal serotype. J Biol Chem 287:27885–27894. doi:10.1074/jbc.M112.380451. 22736767 PMC3431705

[13] Park IH, Geno KA, Yu J, Oliver MB, Kim KH, Nahm MH 2015 Genetic, biochemical, and serological characterization of a new pneumococcal serotype, 6H, and generation of a pneumococcal strain producing three different capsular repeat units. Clin Vaccine Immunol 22:313–318. doi:10.1128/CVI.00647-14. 25589550 PMC4340893

[14] Feikin DR, Kagucia EW, Loo JD, Link-Gelles R, Puhan MA, Cherian T, Levine OS, Whitney CG, O'Brien KL, Moore MR 2013 Serotype-specific changes in invasive pneumococcal disease after pneumococcal conjugate vaccine introduction: a pooled analysis of multiple surveillance sites. PLoS Med 10:e1001517. doi:10.1371/journal.pmed.1001517. 24086113 PMC3782411

[15] Hausdorff WP, Hoet B, Adegbola RA 2015 Predicting the impact of new pneumococcal conjugate vaccines: serotype composition is not enough. Expert Rev Vaccines 14:413–428. doi:10.1586/14760584.2015.965160. 25266168

[16] Weinberger DM, Malley R, Lipsitch M 2011 Serotype replacement in disease after pneumococcal vaccination. Lancet 378:1962–1973. doi:10.1016/S0140-6736(10)62225-8. 21492929 PMC3256741

[17] Brueggemann AB, Pai R, Crook DW, Beall B 2007 Vaccine escape recombinants emerge after pneumococcal vaccination in the United States. PLoS Pathog 3:e168. doi:10.1371/journal.ppat.0030168. 18020702 PMC2077903

[18] Golubchik T, Brueggemann AB, Street T, Gertz RE, Spencer CCA, Ho T, Giannoulatou E, Link-Gelles R, Harding RM, Beall B, Peto TEA, Moore MR, Donnelly P, Crook DW, Bowden R 2012 Pneumococcal genome sequencing tracks a vaccine escape variant formed through a multi-fragment recombination event. Nat Genet 44:352–355. doi:10.1038/ng.1072. 22286217 PMC3303117

[19] Beall BW, Gertz RE, Hulkower RL, Whitney CG, Moore MR, Brueggemann AB 2011 Shifting genetic structure of invasive serotype 19A pneumococci in the United States. J Infect Dis 203:1360–1368. doi:10.1093/infdis/jir052. 21398395 PMC3080895

[20] Yother J 2004 Capsules, p 30–48. *In* Tuomanen E, Mitchell T, Morrison D, Spratt B (ed), The pneumococcus. ASM Press, Washington, DC.

[21] Bentley SD, Aanensen DM, Mavroidi A, Saunders D, Rabbinowitsch E, Collins M, Donohoe K, Harris D, Murphy L, Quail MA, Samuel G, Skovsted IC, Kaltoft MS, Barrell B, Reeves PR, Parkhill J, Spratt BG 2006 Genetic analysis of the capsular biosynthetic locus from all 90 pneumococcal serotypes. PLoS Genet 2:e31. doi:10.1371/journal.pgen.0020031. 16532061 PMC1391919

[22] Coffey TJ, Enright MC, Daniels M, Morona JK, Morona R, Hryniewicz W, Paton JC, Spratt BG 1998 Recombinational exchanges at the capsular polysaccharide biosynthetic locus lead to frequent serotype changes among natural isolates of Streptococcus pneumoniae. Mol Microbiol 27:73–83. doi:10.1046/j.1365-2958.1998.00658.x. 9466257

[23] Coffey TJ, Enright MC, Daniels M, Wilkinson P, Berron S, Fenoll A, Spratt BG 1998 Serotype 19A variants of the Spanish serotype 23F multiresistant clone of Streptococcus pneumoniae. Microb Drug Resist 4:51–55. doi:10.1089/mdr.1998.4.51. 9533725

[24] Coffey TJ, Daniels M, Enright MC, Spratt BG 1999 Serotype 14 variants of the Spanish penicillin-resistant serotype 9V clone of Streptococcus pneumoniae arose by large recombinational replacements of the *cpsA-pbp1a* region. Microbiology 145:2023–2031. doi:10.1099/13500872-145-8-2023. 10463168

[25] Wyres KL, Lambertsen LM, Croucher NJ, McGee L, von Gottberg A, Linares J, Jacobs MR, Kristinsson KG, Beall BW, Klugman KP, Parkhill J, Hakenbeck R, Bentley SD, Brueggemann AB 2013 Pneumococcal capsular switching: a historical perspective. J Infect Dis 207:439–449. doi:10.1093/infdis/jis703. 23175765 PMC3537446

[26] Feikin DR, Klugman KP 2002 Historical changes in pneumococcal serogroup distribution: implications for the era of pneumococcal conjugate vaccines. Clin Infect Dis 35:547–555. doi:10.1086/341896. 12173128

[27] Park IH, Pritchard DG, Cartee R, Brandao A, Brandileone MC, Nahm MH 2007 Discovery of a new capsular serotype (6C) within serogroup 6 of Streptococcus pneumoniae. J Clin Microbiol 45:1225–1233. doi:10.1128/JCM.02199-06. 17267625 PMC1865839

[28] Jin P, Kong F, Xiao M, Oftadeh S, Zhou F, Liu C, Russell F, Gilbert GL 2009 First report of putative Streptococcus pneumoniae serotype 6D among nasopharyngeal isolates from Fijian children. J Infect Dis 200:1375–1380. doi:10.1086/606118. 19803727

[29] Bratcher PE, Kim KH, Kang JH, Hong JY, Nahm MH 2010 Identification of natural pneumococcal isolates expressing serotype 6D by genetic, biochemical and serological characterization. Microbiology 156:555–560. doi:10.1099/mic.0.034116-0. 19942663 PMC2890086

[30] Millar EV, Pimenta FC, Roundtree A, Jackson D, Carvalho Mda G, Perilla MJ, Reid R, Santosham M, Whitney CG, Beall BW, O'Brien KL 2010 Pre- and post-conjugate vaccine epidemiology of pneumococcal serotype 6C invasive disease and carriage within Navajo and White Mountain Apache communities. Clin Infect Dis 51:1258–1265. doi:10.1086/657070. 21034194

[31] Grant LR, O'Brien SE, Burbidge P, Haston M, Zancolli M, Cowell L, Johnson M, Weatherholtz RC, Reid R, Santosham M, O'Brien KL, Goldblatt D 2013 Comparative immunogenicity of 7- and 13-valent pneumococcal conjugate vaccines and the development of functional antibodies to cross-reactive serotypes. PLoS One 8:e74906. doi:10.1371/journal.pone.0074906. 24086394 PMC3781100

[32] Cooper D, Yu X, Sidhu M, Nahm MH, Fernsten P, Jansen KU 2011 The 13-valent pneumococcal conjugate vaccine (PCV13) elicits cross-functional opsonophagocytic killing responses in humans to Streptococcus pneumoniae serotypes 6C and 7A. Vaccine 29:7207–7211. doi:10.1016/j.vaccine.2011.06.056. 21689707 PMC3170457

[33] Vesikari T, Wysocki J, Chevallier B, Karvonen A, Czajka H, Arsene JP, Lommel P, Dieussaert I, Schuerman L 2009 Immunogenicity of the 10-valent pneumococcal non-typeable Haemophilus influenzae protein D conjugate vaccine (PHiD-CV) compared to the licensed 7vCRM vaccine. Pediatr Infect Dis J 28(4 Suppl):S66–S76. doi:10.1097/INF.0b013e318199f8ef. 19325449

[34] Choi EH, Lee HJ, Cho EY, Oh CE, Eun BW, Lee J, Kim MJ 2010 Prevalence and genetic structures of Streptococcus pneumoniae serotype 6D, South Korea. Emerg Infect Dis 16:1751–1753. doi:10.3201/eid1611.100941. 21029535 PMC3294534

[35] Lee H, Cha JH, Nahm MH, Burton RL, Kim KH 2013 The 7-valent pneumococcal conjugate vaccine elicits cross-functional opsonophagocytic killing responses to Streptococcus pneumoniae serotype 6D in children. BMC Infect Dis 13:474. doi:10.1186/1471-2334-13-474. 24112237 PMC3852776

[36] Mavroidi A, Godoy D, Aanensen DM, Robinson DA, Hollingshead SK, Spratt BG 2004 Evolutionary genetics of the capsular locus of serogroup 6 pneumococci. J Bacteriol 186:8181–8192. doi:10.1128/JB.186.24.8181-8192.2004. 15576766 PMC532438

[37] Elberse K, Witteveen S, van der Heide H, van de Pol I, Schot C, van der Ende A, Berbers G, Schouls L 2011 Sequence diversity within the capsular genes of Streptococcus pneumoniae serogroup 6 and 19. PLoS One 6:e25018. doi:10.1371/journal.pone.0025018. 21949837 PMC3174988

[38] Ko KS, Park IH, Baek JY, Song J-H 2013 Capsular gene sequences and genotypes of “serotype 6E” Streptococcus pneumoniae isolates. J Clin Microbiol 51:3395–3399. doi:10.1128/JCM.01645-13. 23824778 PMC3811659

[39] Baek JY, Park IH, So TM, Lalitha MK, Shimono N, Yasin RM, Carlos CC, Perera J, Thamlikitkul V, Hsueh PR, Van PH, Shibl AM, Song JH, Ko KS 2014 Prevalence and characteristics of Streptococcus pneumoniae “putative serotype 6E” isolates from Asian countries. Diagn Microbiol Infect Dis 80:334–337. doi:10.1016/j.diagmicrobio.2014.08.017. 25439447

[40] Kawaguchiya M, Urushibara N, Kobayashi N 2015 High prevalence of genotype 6E (putative serotype 6E) among noninvasive/colonization isolates of Streptococcus pneumoniae in northern Japan. Microb Drug Resist 21:209–214. doi:10.1089/mdr.2014.0181. 25361198

[41] Chewapreecha C, Harris SR, Croucher NJ, Turner C, Marttinen P, Cheng L, Pessia A, Aanensen DM, Mather AE, Page AJ, Salter SJ, Harris D, Nosten F, Goldblatt D, Corander J, Parkhill J, Turner P, Bentley SD 2014 Dense genomic sampling identifies highways of pneumococcal recombination. Nat Genet 46:305–309. doi:10.1038/ng.2895. 24509479 PMC3970364

[42] Croucher NJ, Finkelstein JA, Pelton SI, Mitchell PK, Lee GM, Parkhill J, Bentley SD, Hanage WP, Lipsitch M 2013 Population genomics of post-vaccine changes in pneumococcal epidemiology. Nat Genet 45:656–663. doi:10.1038/ng.2625. 23644493 PMC3725542

[43] Croucher NJ, Hanage WP, Harris SR, McGee L, van der Linden M, de Lencastre H, Sa-Leao R, Song JH, Ko KS, Beall B, Klugman KP, Parkhill J, Tomasz A, Kristinsson KG, Bentley SD 2014 Variable recombination dynamics during the emergence, transmission and ‘disarming’ of a multidrug-resistant pneumococcal clone. BMC Biol 12:49. doi:10.1186/1741-7007-12-49. 24957517 PMC4094930

[44] Wyres KL, Lambertsen LM, Croucher NJ, McGee L, von Gottberg A, Linares J, Jacobs MR, Kristinsson KG, Beall BW, Klugman KP, Parkhill J, Hakenbeck R, Bentley SD, Brueggemann AB 2012 The multidrug-resistant PMEN1 pneumococcus is a paradigm for genetic success. Genome Biol 13:R103. doi:10.1186/gb-2012-13-11-r103. 23158461 PMC3580495

[45] Wyres KL, van Tonder A, Lambertsen LM, Hakenbeck R, Parkhill J, Bentley SD, Brueggemann AB 2013 Evidence of antimicrobial resistance-conferring genetic elements among pneumococci isolated prior to 1974. BMC Genomics 14:500. doi:10.1186/1471-2164-14-500. 23879707 PMC3726389

[46] van Tonder A, Mistry S, Bray JE, Hill DMC, Cody AJ, Farmer CL, Klugman KP, von Gottberg A, Bentley SD, Parkhill J, Jolley KA, Maiden MCJ, Brueggemann AB 2014 Defining the estimated core genome of bacterial populations using a Bayesian decision model. PLoS Comput Biol 10:e1003788. doi:10.1371/journal.pcbi.1003788. 25144616 PMC4140633

[47] Croucher NJ, Harris SR, Fraser C, Quail MA, Burton J, van der Linden M, McGee L, von Gottberg A, Song JH, Ko KS, Pichon B, Baker S, Parry CM, Lambertsen LM, Shahinas D, Pillai DR, Mitchell TJ, Dougan G, Tomasz A, Klugman KP, Parkhill J, Hanage WP, Bentley SD 2011 Rapid pneumococcal evolution in response to clinical interventions. Science 331:430–434. doi:10.1126/science.1198545. 21273480 PMC3648787

[48] McGee L, McDougal L, Zhou J, Spratt BG, Tenover FC, George R, Hakenbeck R, Hryniewicz W, Lefévre JC, Tomasz A, Klugman KP 2001 Nomenclature of major antimicrobial-resistant clones of Streptococcus pneumoniae defined by the Pneumococcal Molecular Epidemiology Network. J Clin Microbiol 39:2565–2571. doi:10.1128/JCM.39.7.2565-2571.2001. 11427569 PMC88185

[49] Zerbino DR, Birney E 2008 Velvet: algorithms for *de novo* short read assembly using de Bruijn graphs. Genome Res 18:821–829. doi:10.1101/gr.074492.107. 18349386 PMC2336801

[50] Jolley KA, Maiden MC 2010 BIGSdb: scalable analysis of bacterial genome variation at the population level. BMC Bioinformatics 11:595. doi:10.1186/1471-2105-11-595. 21143983 PMC3004885

[51] Jolley KA, Bliss CM, Bennett JS, Bratcher HB, Brehony C, Colles FM, Wimalarathna H, Harrison OB, Sheppard SK, Cody AJ, Maiden MC 2012 Ribosomal multilocus sequence typing: universal characterization of bacteria from domain to strain. Microbiology 158:1005–1015. doi:10.1099/mic.0.055459-0. 22282518 PMC3492749

[52] Li W, Godzik A 2006 Cd-hit: a fast program for clustering and comparing large sets of protein or nucleotide sequences. Bioinformatics 22:1658–1659. doi:10.1093/bioinformatics/btl158. 16731699

[53] Park IH, Park S, Hollingshead SK, Nahm MH 2007 Genetic basis for the new pneumococcal serotype, 6C. Infect Immun 75:4482–4489. doi:10.1128/IAI.00510-07. 17576753 PMC1951153

[54] Altschul SF, Madden TL, Schäffer AA, Zhang J, Zhang Z, Miller W, Lipman DJ 1997 Gapped BLAST and PSI-BLAST: a new generation of protein database search programs. Nucleic Acids Res 25:3389–3402. doi:10.1093/nar/25.17.3389. 9254694 PMC146917

[55] Edgar RC 2004 MUSCLE: multiple sequence alignment with high accuracy and high throughput. Nucleic Acids Res 32:1792–1797. doi:10.1093/nar/gkh340. 15034147 PMC390337

[56] Price MN, Dehal PS, Arkin AP 2010 FastTree 2—approximately maximum-likelihood trees for large alignments. PLoS One 5:e9490. doi:10.1371/journal.pone.0009490. 20224823 PMC2835736

[57] Letunic I, Bork P 2011 Interactive Tree Of Life v2: online annotation and display of phylogenetic trees made easy. Nucleic Acids Res 39:W475–W478. doi:10.1093/nar/gkr201. 21470960 PMC3125724

[58] Tamura K, Peterson D, Peterson N, Stecher G, Nei M, Kumar S 2011 MEGA5: molecular evolutionary genetics analysis using maximum likelihood, evolutionary distance, and maximum parsimony methods. Mol Biol Evol 28:2731–2739. doi:10.1093/molbev/msr121. 21546353 PMC3203626

[59] Didelot X, Wilson DJ 2015 ClonalFrameML: efficient inference of recombination in whole bacterial genomes. PLoS Comput Biol 11:e1004041. doi:10.1371/journal.pcbi.1004041. 25675341 PMC4326465

[60] Francisco AP, Vaz C, Monteiro PT, Melo-Cristino J, Ramirez M, Carrico JA 2012 PHYLOViZ: phylogenetic inference and data visualization for sequence based typing methods. BMC Bioinformatics 13:87. doi:10.1186/1471-2105-13-87. 22568821 PMC3403920

[61] Findlow H, Borrow R, Andrews N, Waight P, Sheasby E, Matheson M, England A, Goldblatt D, Ashton L, Findlow J, Miller E 2012 Immunogenicity of a single dose of meningococcal group C conjugate vaccine given at 3 months of age to healthy infants in the United kingdom. Pediatr Infect Dis J 31:616–622. doi:10.1097/INF.0b013e31824f34e6. 22333698

[62] Andrews NJ, Waight PA, Burbidge P, Pearce E, Roalfe L, Zancolli M, Slack M, Ladhani SN, Miller E, Goldblatt D 2014 Serotype-specific effectiveness and correlates of protection for the 13-valent pneumococcal conjugate vaccine: a postlicensure indirect cohort study. Lancet Infect Dis 14:839–846. doi:10.1016/S1473-3099(14)70822-9. 25042756

[63] Burton RL, Nahm MH 2012 Development of a fourfold multiplexed opsonophagocytosis assay for pneumococcal antibodies against additional serotypes and discovery of serological subtypes in Streptococcus pneumoniae serotype 20. Clin Vaccine Immunol 19:835–841. doi:10.1128/CVI.00086-12. 22518015 PMC3370448

[64] Turner P, Turner C, Jankhot A, Helen N, Lee SJ, Day NP, White NJ, Nosten F, Goldblatt D 2012 A longitudinal study of Streptococcus pneumoniae carriage in a cohort of infants and their mothers on the Thailand-Myanmar border. PLoS One 7:e38271. doi:10.1371/journal.pone.0038271. 22693610 PMC3365031

