# Supplementary material for: Genomics Reveals the Worldwide Distribution of Multidrug-Resistant Serotype 6E Pneumococci
Source: J Clin Microbiol. 2015 Jun 18;53(7):2271–85. doi: 10.1128/JCM.00744-15 (PMC4473186; doi:10.1128/JCM.00744-15)
Supplement: Supplemental material [file supp_53_7_2271__index.html]

Supplemental material 

# Genomics reveals the worldwide distribution of multidrug-resistant serotype 6E pneumococci

## Supplemental material

- Supplemental file 1 -

  Table S1 (Entire data set of 974 genomes)

  XLSX, 129K
- Supplemental file 2 -

  Fig. S1 (Description of the sequence-based serotyping pipeline)

  PDF, 43K
- Supplemental file 3 -

  Fig. S2 (Phylogenetic trees for each of the 13 common genes in the *cps* locus, constructed using the entire set of 974 genomes)

  PDF, 2.2M
- Supplemental file 4 -

  Fig. S3 (Comparison, based on the entire data set of 974 genomes, of the original serotyping data as previously published for each genome versus the sequence-based serotyping data generated in this study)

  PDF, 165K
- Supplemental file 5 -

  Fig. S4 (Gene-by-gene depiction of the variable amino acids for all pneumococci that demonstrated evidence for capsular switching)

  PDF, 5.7M
- Supplemental file 6 -

  Legends to Fig. S1 to S4

  PDF, 75K
