# Supplementary figures and images for: Genomics Reveals the Worldwide Distribution of Multidrug-Resistant Serotype 6E Pneumococci
Source: J Clin Microbiol. 2015 Jun 18;53(7):2271–85. doi: 10.1128/JCM.00744-15 (PMC4473186; doi:10.1128/JCM.00744-15)

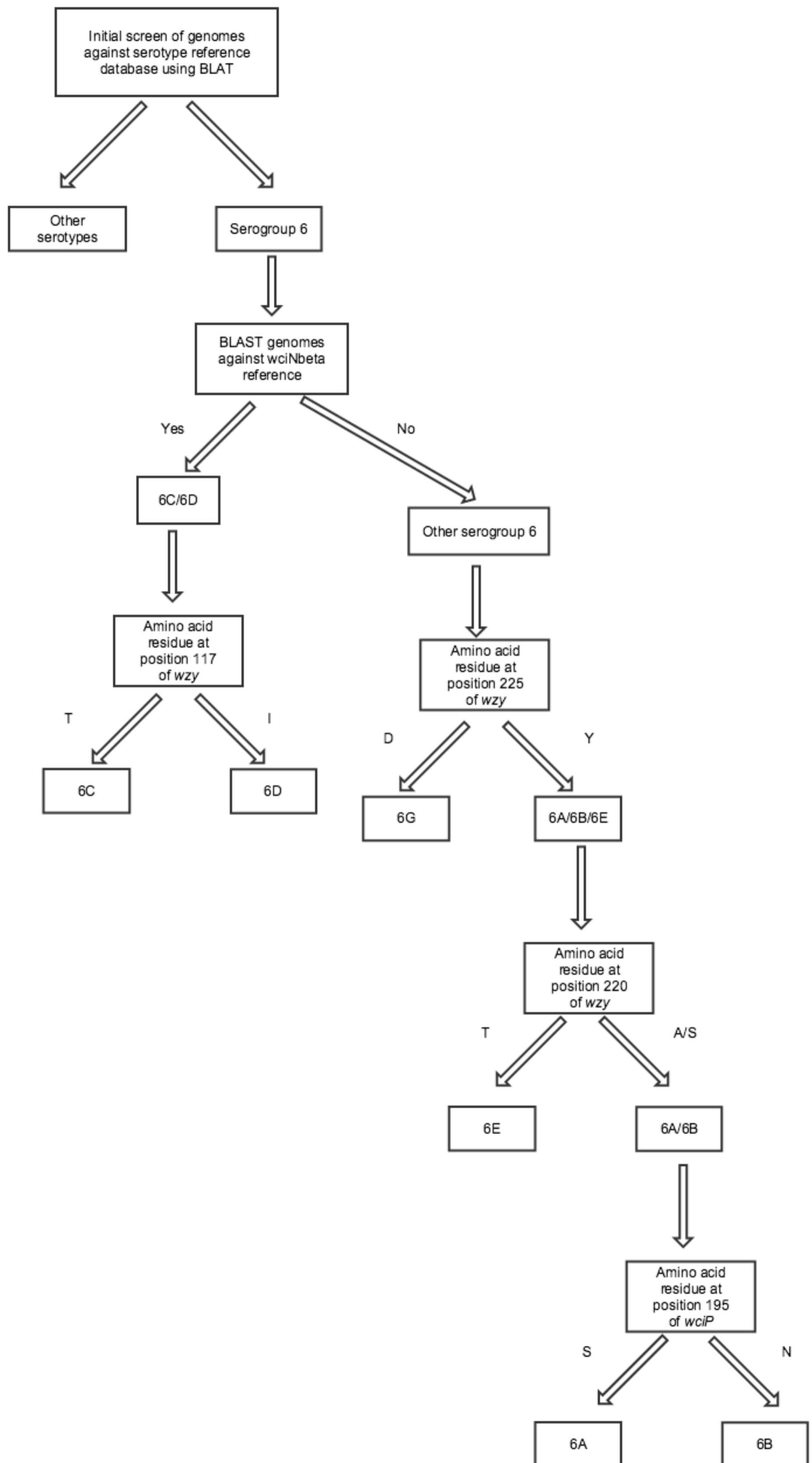

Supplement: Supplemental material [file JCM.00744-15_zjm999094368so2.pdf]

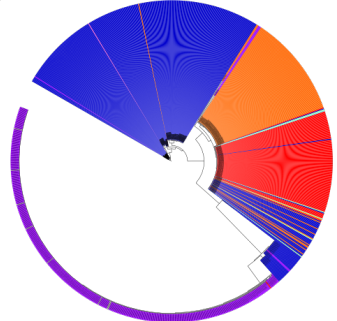

wzg

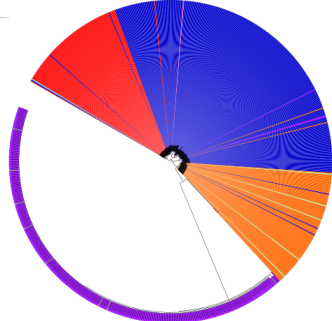

wzh

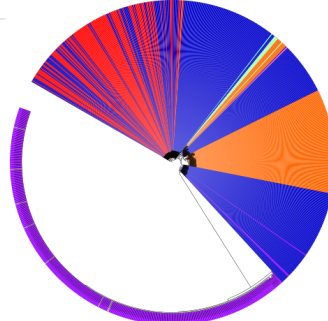

wzd

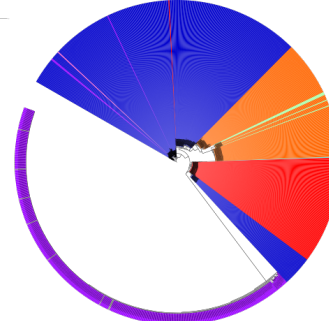

wze

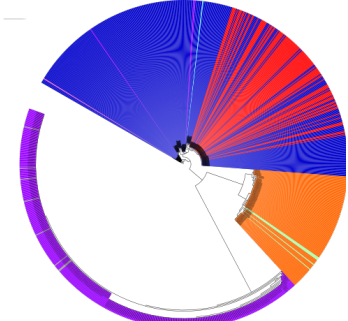

wchA

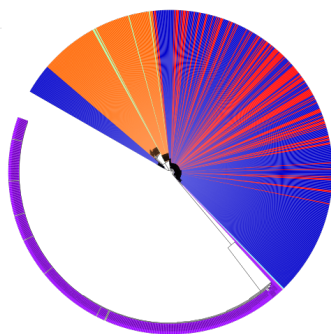

wciO

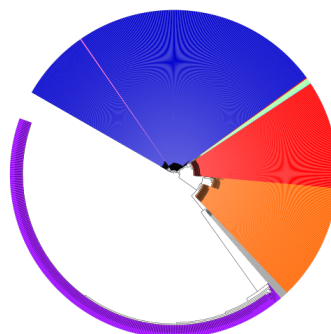

wciP

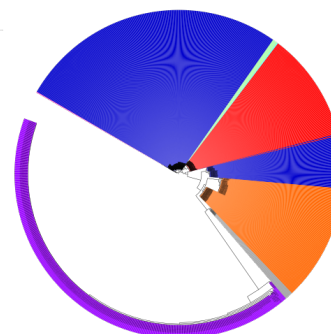

wzy

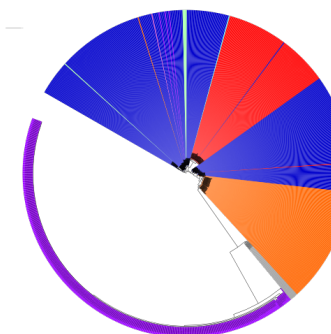

wzx

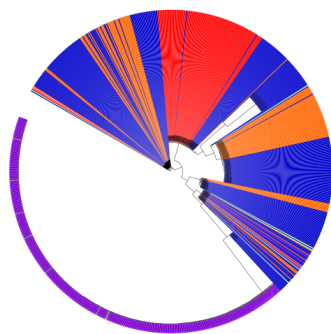

rmlA

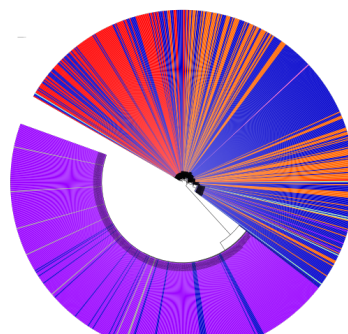

rmlC

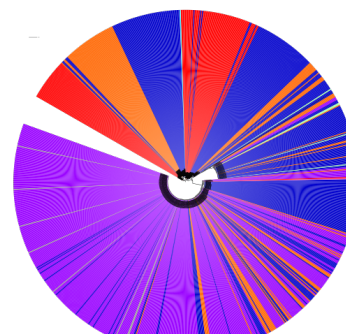

rmlB

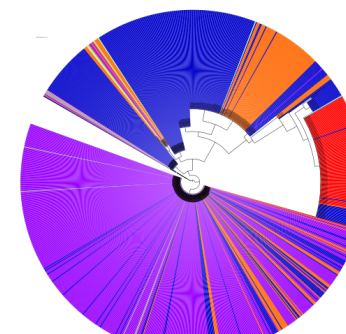

rmlD

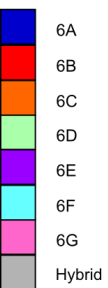

Supplement: Supplemental material [file JCM.00744-15_zjm999094368so3.pdf]

A

Original serotype data

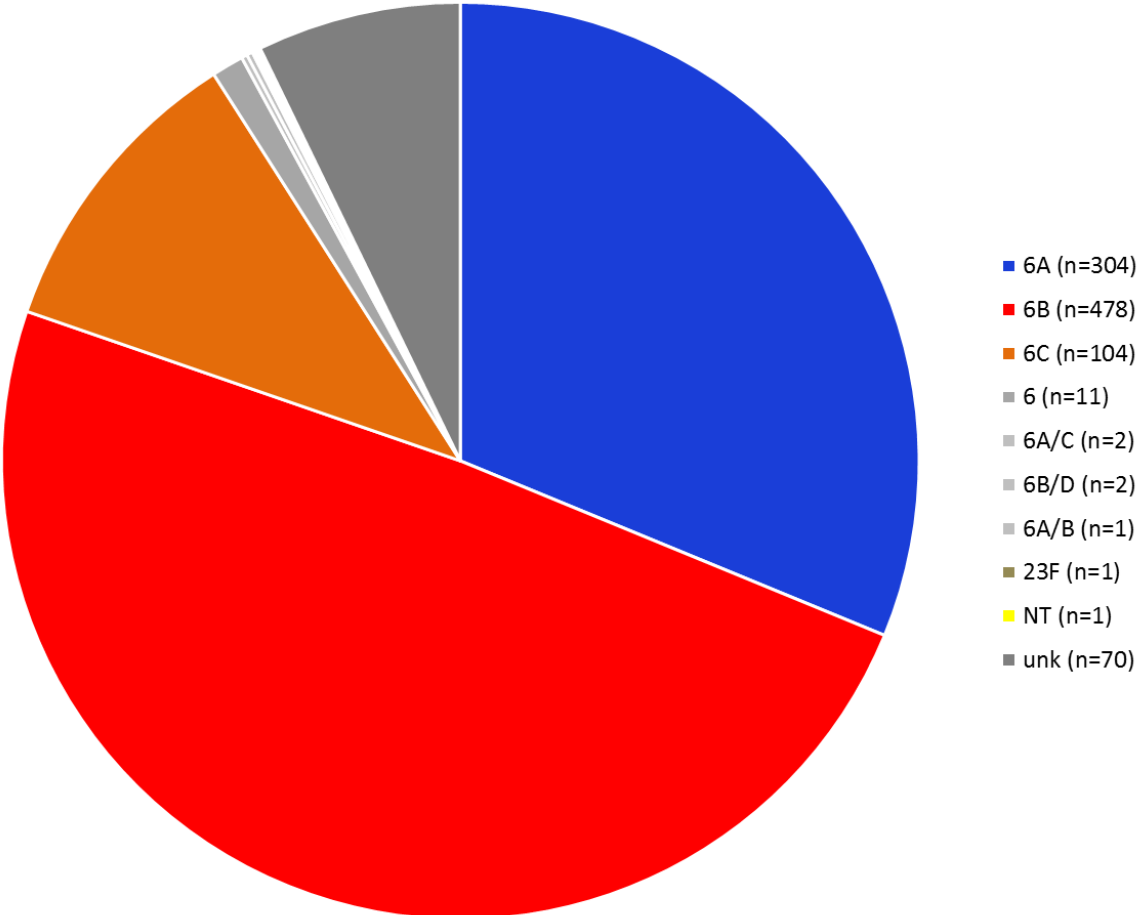

B

Sequence-based serotype data

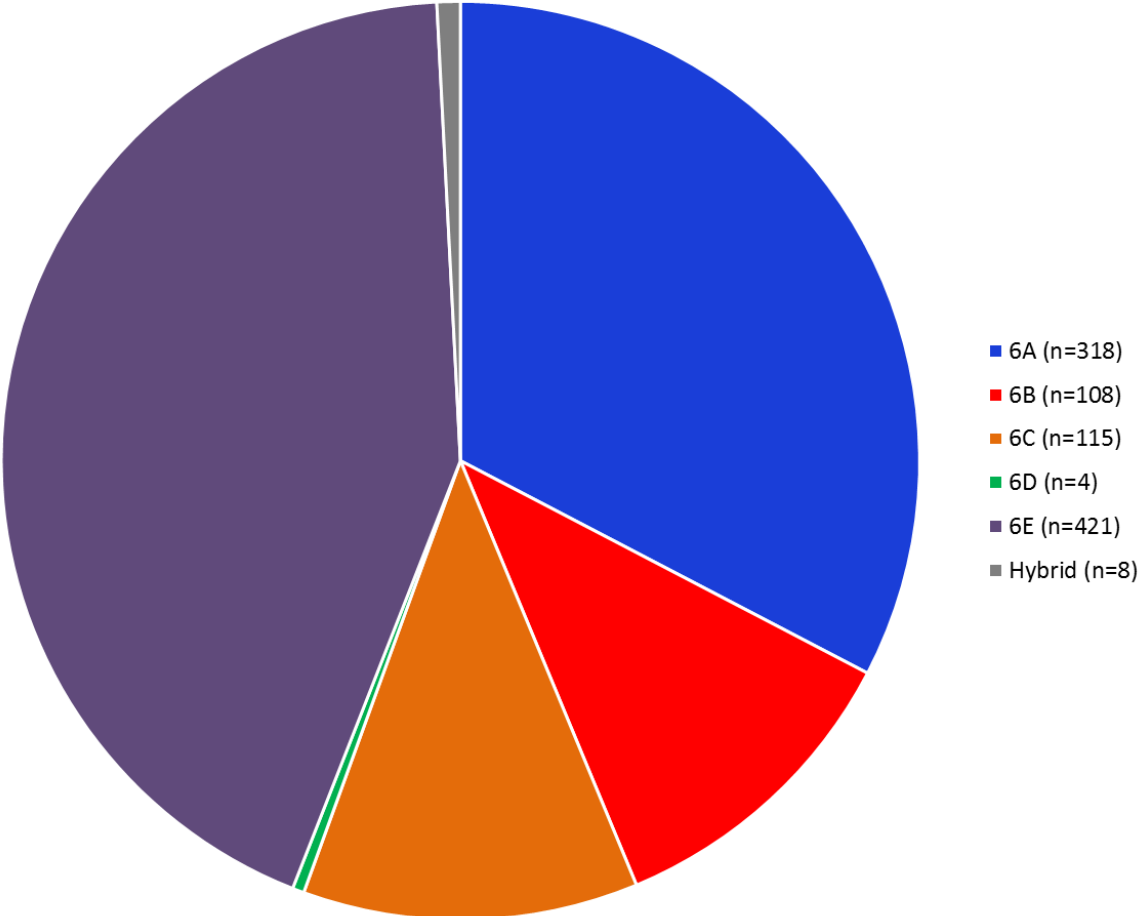

Supplement: Supplemental material [file JCM.00744-15_zjm999094368so4.pdf]

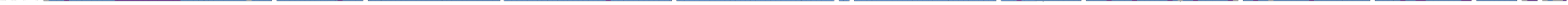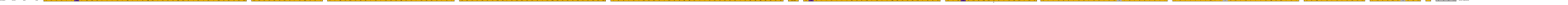

Supplement: Supplemental material [file JCM.00744-15_zjm999094368so5.pdf]
