# Supplementary material for: Genomics Reveals the Worldwide Distribution of Multidrug-Resistant Serotype 6E Pneumococci
Source: J Clin Microbiol. 2015 Jun 18;53(7):2271–85. doi: 10.1128/JCM.00744-15 (PMC4473186; doi:10.1128/JCM.00744-15)
Supplement: Supplemental material [file JCM.00744-15_zjm999094368so6.pdf]

SUPPLEMENTARY FIGURE LEGENDS

Supplementary Figure 1: Description of the sequence-based serotyping pipeline.

Supplementary Figure 2: Phylogenetic trees for each of the 13 common genes in the *cps* locus, constructed using the entire set of 974 genomes.

Supplementary Figure 3: A comparison of the original serotyping data as previously published for each genome (A) versus the sequence-based serotyping data generated in this study (B), using the entire dataset of 974 genomes. The unknown serotypes in part A correspond to Icelandic pneumococci from the study currently in progress and for which serotyping data generated via traditional methods were not available.

Supplementary Figure 4: Gene-by-gene depiction of the variable amino acids for all pneumococci that demonstrated evidence for capsular switching. The appropriate serotype reference strains for each comparison are at the top of each section (see Table 1 for accession numbers). Residues were coloured by serotype: 6A (blue), 6B (red), 6C (orange), 6D (pale green), and other (grey). Numbers above each variable residue mark the location of that residue in that gene.
